# Transcatheter aortic valve replacement via direct aortic access for severe aortic stenosis with double aortic arch

**DOI:** 10.1093/jscr/rjaf251

**Published:** 2025-04-22

**Authors:** Akihito Arai, Mimiko Tabata, Kenichiro Takahashi, Ryo Izubuchi, Minako Hayakawa, Joshi Tsutsumi, Akihiro Urabe

**Affiliations:** Department of Cardiovascular Surgery, Yamato Seiwa Hospital, 9-8-2 Minami-rinkan, Yamato-shi 242-0006, Kanagawa, Japan; Department of Cardiovascular Surgery, Yamato Seiwa Hospital, 9-8-2 Minami-rinkan, Yamato-shi 242-0006, Kanagawa, Japan; Department of Cardiovascular Surgery, Yamato Seiwa Hospital, 9-8-2 Minami-rinkan, Yamato-shi 242-0006, Kanagawa, Japan; Department of Cardiovascular Surgery, Yamato Seiwa Hospital, 9-8-2 Minami-rinkan, Yamato-shi 242-0006, Kanagawa, Japan; Department of Cardiovascular Surgery, Yamato Seiwa Hospital, 9-8-2 Minami-rinkan, Yamato-shi 242-0006, Kanagawa, Japan; Department of Cardiology, Yamato Seiwa Hospital, 9-8-2 Minami-rinkan, Yamato-shi 242-0006, Kanagawa, Japan; Department of Cardiology, Yamato Seiwa Hospital, 9-8-2 Minami-rinkan, Yamato-shi 242-0006, Kanagawa, Japan

**Keywords:** transcatheter aortic valve replacement, double aortic arch, direct aortic approach

## Abstract

Transcatheter aortic valve replacement (TAVR) is an established treatment for managing severe aortic stenosis. Preoperative planning requires cautious identification of the access route, which can be challenging in patients with anatomical abnormalities of the aorta. Double aortic arch (DAA) is a congenital condition where the aorta bifurcates into two separate vessels that encircle the trachea and esophagus, thereby forming a vascular ring. This condition accounts for ~1% of congenital cardiovascular anomalies. Previous reports on TAVR performed on patients with DAA are limited, and there is no consensus on the appropriate access route. Herein, we present an 85-year-old female patient with DAA and aortic stenosis who underwent a successful TAVR using the direct aortic approach.

## Introduction

Transcatheter aortic valve replacement (TAVR) is an established treatment for severe aortic stenosis in elderly patients and those with comorbidities. TAVR is a relatively safe procedure, and the risk of significant vascular damage in TAVR has decreased with lower-profile valve delivery systems. However, the incidence of vascular complications remains at ~5%–8% [1]. The common risk factors of vascular complications during TAVR are peripheral vascular disease, severe calcification, and tortuosity of the arterial system [2]. Thus, preoperative planning requires cautious identification of the access route. Double aortic arch (DAA), a rare congenital anomaly, can occasionally present with significant aortic tortuosity. Aging and subsequent aortic elongation in such congenital conditions can accentuate aortic tortuosity, thereby forming a complex route via the femoral artery. The incidence of vascular complication associated with TAVR is unknown. Consequently, TAVR requires more vigilant assessment of the access route in patients with coexisting anatomic abnormalities.

Herein, we report a case of successful TAVR using the direct aortic approach in a patient with DAA and severe tortuosity of the descending aorta.

## Case

**Figure 1:**
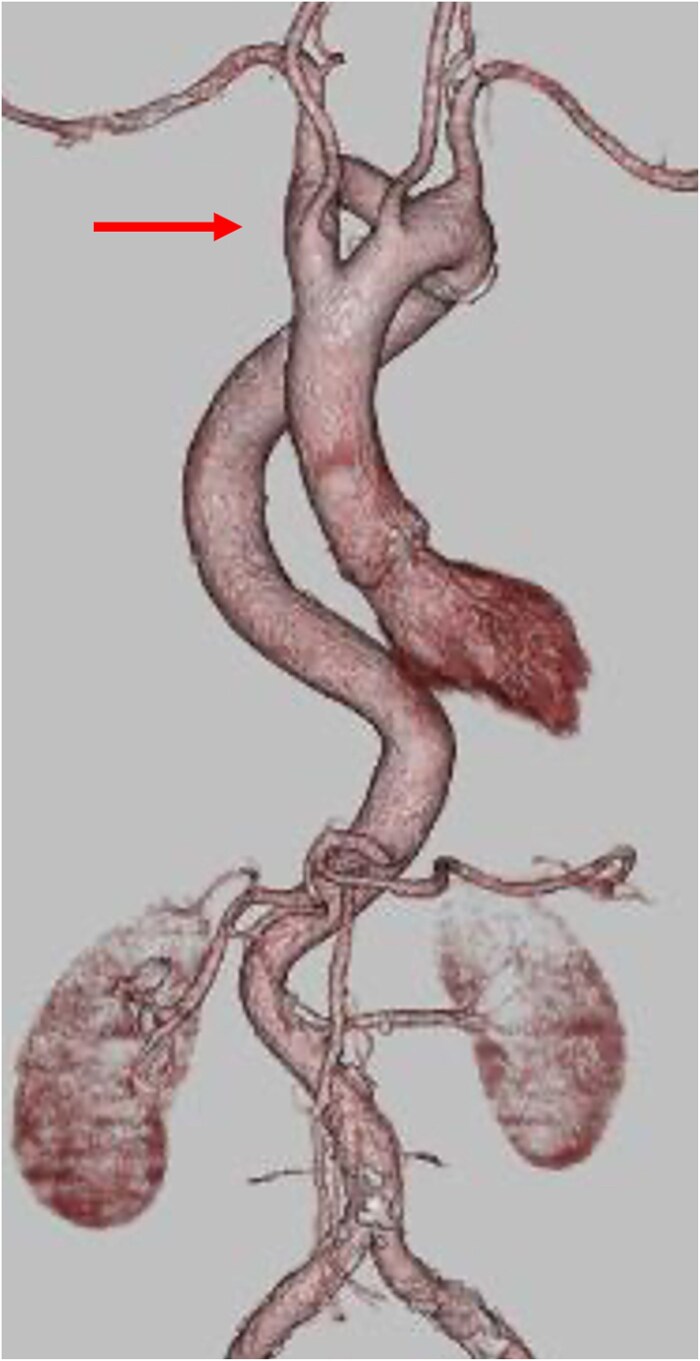
Three-dimensional CT reconstruction showing the double aortic arch (arrow) compressing the trachea.

An 85-year-old female patient with a history of left breast cancer surgery was referred to our institution due to severe aortic stenosis. Transthoracic echocardiogram revealed the presence of a severely calcified aortic valve, with a peak velocity of 4.0 m/s, mean pressure gradient of 27.8 mmHg, and valve area of 0.72 cm^2^. Computed tomography scan showed a right dominant DAA with both aortic arches surrounding the trachea and esophagus, thereby forming a complete vascular ring (Fig. 1 and Video 1). In addition, the descending aorta was located on the right side of the thoracic cavity and returned to the left side at the level of the diaphragm. This phenomenon caused an abnormal aortic tortuosity.

**Figure 2:**
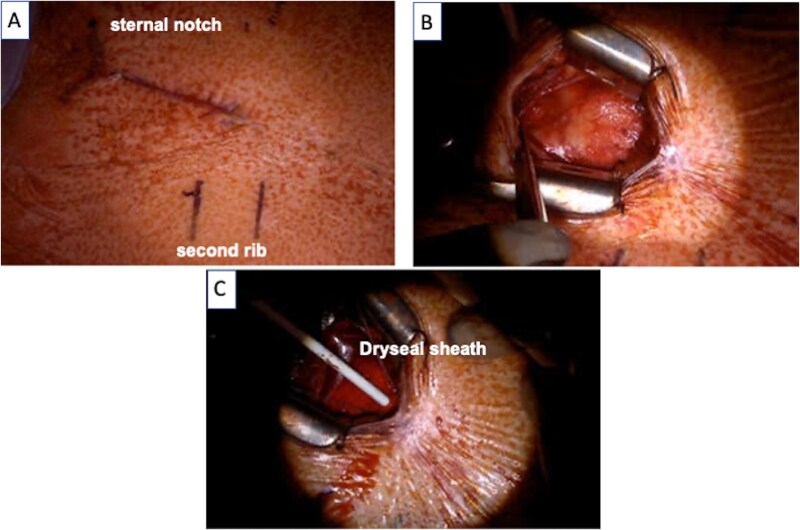
(A) A skin incision was made 5 cm from the sternal notch to the upper edge of the second rib. (B) The ascending aorta was exposed via J-shape partial sternotomy. (C) Evolut PRO PLUS 29 mm valve was inserted via the preinserted Dryseal sheath.

Considering the patient’s age, TAVR was indicated. The anatomical complexity of the arch and descending aorta was a risk factor of intraoperative vascular complication caused by the trans-femoral and trans-subclavian approach. Accordingly, the direct aortic approach was selected. During surgery, the ascending aorta was exposed via J-shape partial sternotomy (Fig. 2A and B), and the access site was secured with a purse-string suture. Evolut PRO PLUS 29 mm valve (Medtronic, Minneapolis, Minnesota) was inserted via the preinserted DrySeal Flex Introducer Sheath (20 Fr) (W.L. Gore & Associates, Newark, Delaware), and the valve was implanted (Fig. 2C). The postoperative course was uneventful.

## Discussion

A vascular ring is a rare congenital anomaly, accounting for ~1% of congenital cardiovascular anomalies. In this condition, the aortic arch and its branches either completely or incompletely encircle or compress the trachea or esophagus or both [3]. Considering the rarity of vascular ring caused by DAA, data on the management and outcomes of TAVR in patients with this congenital anomaly are limited [4].

In TAVR, the risk of complication must be decreased. Arterial tortuosity is a risk factor of access route complications [2]. In the current case, the complexity of the route via the descending aorta was emphasized due to DAA and ring formation. TAVR includes stretching and traction of the aorta, which can subsequently cause specific stress on junctions and branch points. Therefore, the trans-femoral approach was not appropriate in the current case.

When considering the trans-subclavian approach, there was a concern that the left subclavian artery had areas with a diameter of 6 mm only. Hence, device delivery can be challenging. In addition, the histological stability of the tissues along the access route, including the double aortic arch, remained questionable. In ~15% of patients, DAA is associated with Kommerell diverticulum [5]. Therefore, there are concerns about the potential vulnerability of the diverticulum and surrounding tissues [6]. Moreover, in the event of an aortic dissection, the anatomical complexity can make the surgery itself more complicated and increase the risk of complications. Due to these reasons, the trans-subclavian approach was not considered.

In the direct aortic approach, the pathway to the aortic valve is linear, which can eliminate stress on junctions and branch points in the aortic arch. In addition, insertion of the deployment system via the Dryseal sheath was useful for preventing friction to the aortic wall during the procedure. The approach, aiming to inhibit potential vascular complications such as aortic dissection and rupture, which require complex repair due to the underlying anomaly, was selected based on the patient’s anatomical characteristics.

In conclusion, TAVR using the direct aortic approach is associated with reduced risk of potential vascular complications. Hence, it can be an effective alternative for patients with DAA.

## Supplementary Material

DAA_movie_rjaf251

## Data Availability

The data underlying this article will be shared upon reasonable request from the corresponding author.

## References

[1] Aldalati O, MacCarthy P, Dworakowski R, et al. Trans-catheter aortic valve implantation: contemporary practice and the future. Cardiol J 2017;24:206–15. 10.5603/CJ.a2017.0022. 28248406

[2] Mach M, Okutucu S, Kerbel T, et al. Vascular complications in TAVR: incidence, clinical impact, and management. J Clin Med 2021;10:20211028. 10.3390/jcm10215046. PMC8584339 34768565

[3] Hanneman K, Newman B, Chan F. Congenital variants and anomalies of the aortic arch. Radiographics 2017;37:32–51. 10.1148/rg.2017160033. 27860551

[4] Patel RD, Ghadiam HR, Desai AR, et al. Successful transfemoral transcatheter aortic valve replacement in a patient with double aortic arch: an interesting imaging case. Cardiology 2021;146:85–7. 10.1159/000509930. 32957102

[5] Backer CL, Bharadwaj SN, Eltayeb OM, et al. Double aortic arch with Kommerell diverticulum. Ann Thorac Surg 2019;108:161–6. 10.1016/j.athoracsur.2019.01.062. 30849335

[6] Kim KM, Cambria RP, Isselbacher EM, et al. Contemporary surgical approaches and outcomes in adults with Kommerell diverticulum. Ann Thorac Surg 2014;98:1347–54. 10.1016/j.athoracsur.2014.05.045. 25134861

